# Supplementary material for: Antiplatelet Therapy Improves the Prognosis of Patients with Hepatocellular Carcinoma
Source: Cancers (Basel). 2020 Oct 31;12(11):3215. doi: 10.3390/cancers12113215 (PMC7693153; doi:10.3390/cancers12113215)
Supplement: Supplementary file 1 [file cancers-12-03215-s001.pdf]

cancers

MDPI

Supplementary Materials: Antiplatelet Therapy Improves the Prognosis of Patients with Hepatocellular Carcinoma

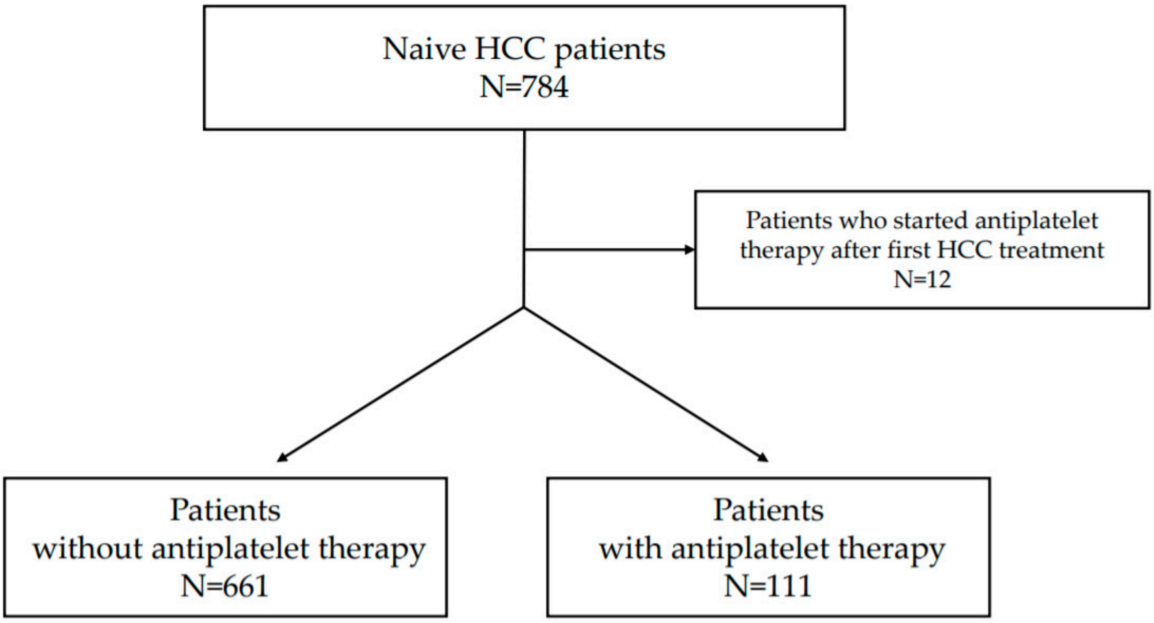

Figure S1. Patients with HCC who entered in this study.

Table S1. Baseline characteristics in HCC patients undertaking or not undertaking antiplatelet therapy after propensity score matching.

	antiplatelet therapy (n=93)	no antiplatelet therapy (n=93)	p-value
Age (IQR), years	76 (68-81)	76 (69-80)	0.69
Male (%)	84.9	59.1	<0.001
Etiology, HBV/HCV/ NBNC	14/43/36	8/51/34	0.30
Child-Pugh grade (A/B/C)	80/11/2	72/21/0	0.06
Stage, I/II/III/IVa/IVb	18/48/22/2/3	14/52/21/2/4	0.95
Tumor number (IQR)	1 (1-2)	1 (1-2)	0.34
Tumor size (IQR), cm	3.2 (2.0-5.0)	2.9 (2.0-5.3)	0.64
Vascular invasion (%)	5.4	4.3	1.00
Extrahepatic Metastasis (%)	3.2	4.3	1.00
AFP (IQR), ng/mL	7.5 (4.5-27.1)	9.5 (4.9-79.9)	0.24
DCP (IQR), mAU/mL	81 (26-526)	63 (22-1174)	0.88
First treatment, curative/TACE/others/BSC	34/56/1/2	33/56/2/2	1.00

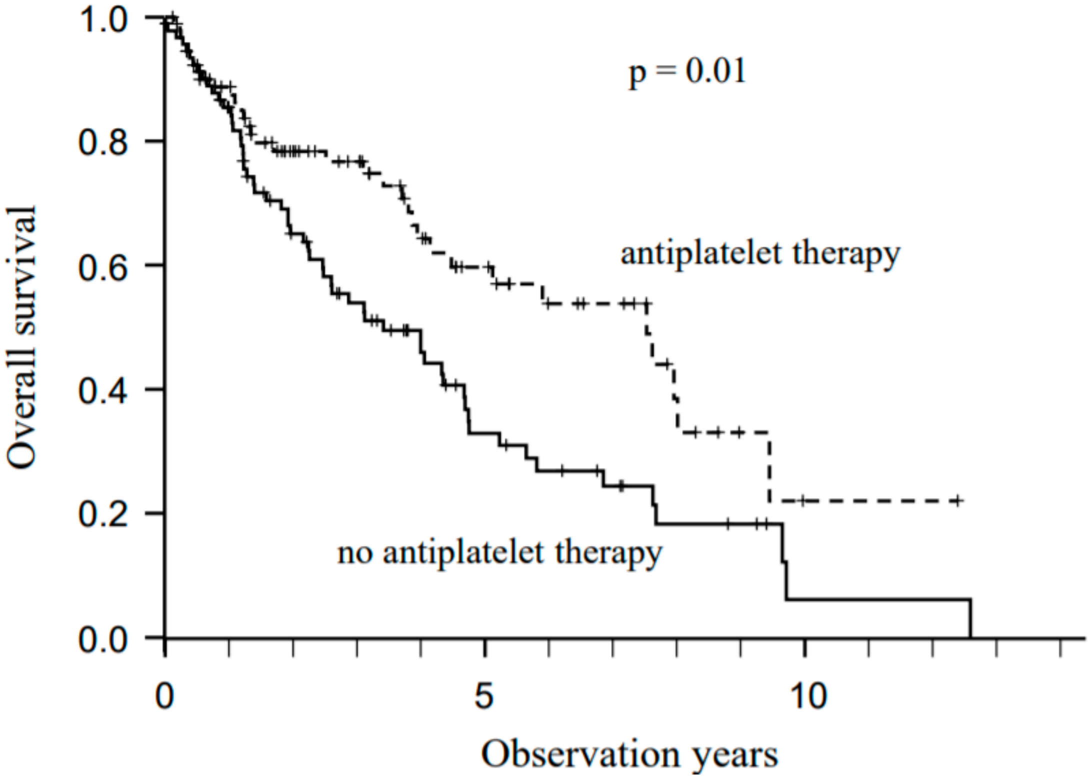

Figure S2. Antiplatelet therapy was associated with better prognosis after propensity score matching. Five-year survival in patients with antiplatelet therapy was significantly better than that without antiplatelet therapy (59.7% versus 32.9%, $p = 0.01$).

© 2020 by the authors. Submitted for possible open access publication under the terms and conditions of the Creative Commons Attribution (CC BY) license (http://creativecommons.org/licenses/by/4.0/).
